# Foreign Body causing Displacement of Immature Fractured Apical Root Fragment: An Unusual Case Report

**DOI:** 10.5005/jp-journals-10005-1520

**Published:** 2018-06-01

**Authors:** Aman Moda, Rajesh Singla, Preeti M Agrawal

**Affiliations:** 1 Ex-Professor, Department of Pedodontics and Preventive Dentistry, Goenka Research Institute of Dental Science, Gandhinagar, Gujarat India; 2 Ex-Postgraduate Student, Department of Pedodontics and Preventive Dentistry Government Dental College, Rohtak, Haryana, India; 3 Professor and Head, Department of Periodontics, Triveni Institute of Dental Sciences Hospital & Research Centre, Bilaspur, Chhattisgarh, India

**Keywords:** Displaced root fragment, Foreign body, Root canal blockage, Root fracture.

## Abstract

Trauma is a common cause of fractured teeth with exposed canals in growing children. These children use foreign bodies like stapler pin, lead pencil, nail, etc., to explore the canal of fractured tooth. Sometimes, these foreign objects may get stuck in the canal, which the children do not reveal to their parents because of fear. These foreign objects may act as a potential source of infection. We herewith present a case of a 12-year-old boy who presented with a stick lodged in the root canal of maxillary right lateral incisor along with the displaced fractured tooth segment at the apex and the associated management.

**How to cite this article:** Moda A, Singla R, Agrawal PM. Foreign Body causing Displacement of Immature Fractured Apical Root Fragment: An Unusual Case Report. Int J Clin Pediatr Dent 2018;11(3):247-249.

## INTRODUCTION

Blockage of the root canal by the foreign body like staple pin,^1^ paper clip,^2^ nail,^3^ metal screw,^4^ etc., generally happens when there is open pulp chamber either due to caries or trauma. This usually happens when a patient tries to remove the food debris but cannot retrieve it back. These objects act as a source of infection and also prevent the clinician from performing proper root canal therapy. Although no separate method is advocated, the methods used for broken files are used for removal of foreign body. These methods include use of fine forceps, endodontic files, ultrasonic instruments, etc.^5^ In case a foreign body is extending beyond apex, then a surgical approach may be necessary for retrieval.^6^

## CASE REPORT

**Fig. 1:**
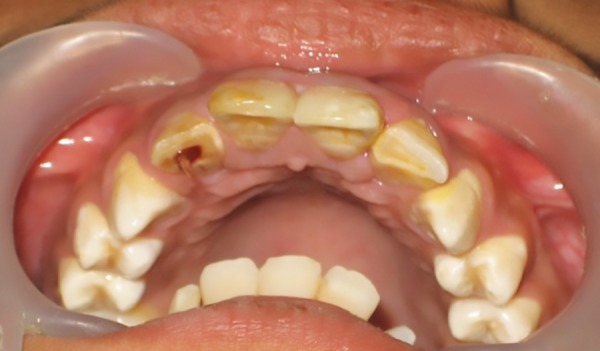
Intraoral view showing Ellis class III fracture of right maxillary lateral incisor

**Fig. 2:**
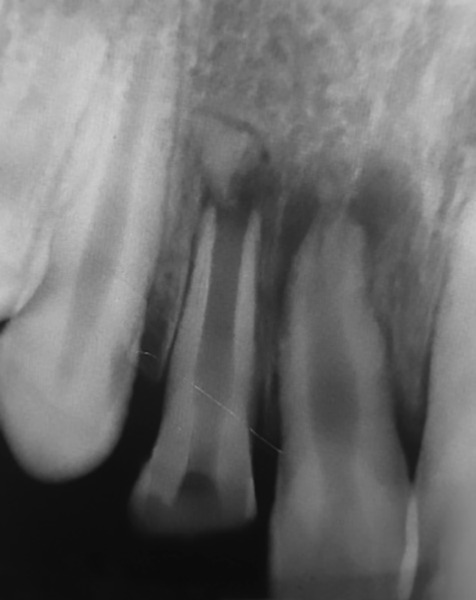
Intraoral periapical radiograph showing class III fracture with displaced apex and periapical radiolucency

A 12-year-old boy presented to the Department of Pedo-dontics with a complaint of broken front tooth and pus discharge. He gave a history of fall while playing 1 year back. Clinical examination revealed Ellis class III fracture of maxillary right lateral incisor (12) (Fig. 1). The tooth was tender to percussion and there was grade I mobility. Radiographic examination revealed displaced fractured root apex with periapical radiolucency (Fig. 2).

**Fig. 3:**
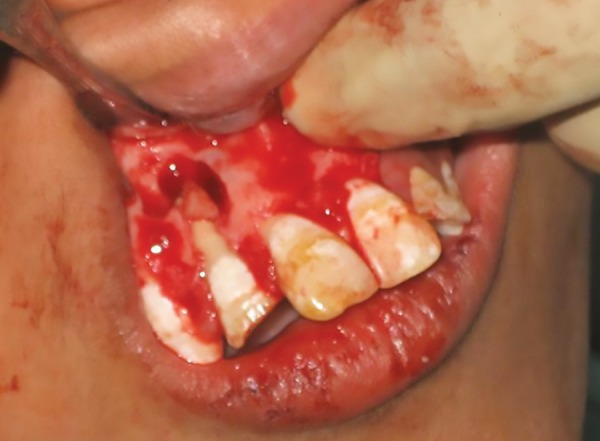
Flap raised with bony window showing fractured root apex

**Fig. 4:**
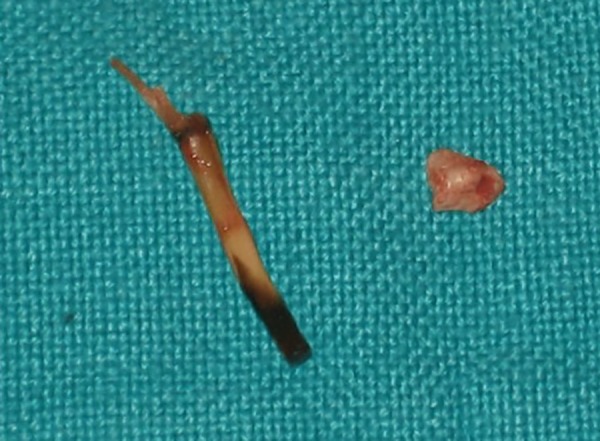
Removal of root apex along with wooden stick

**Fig. 5:**
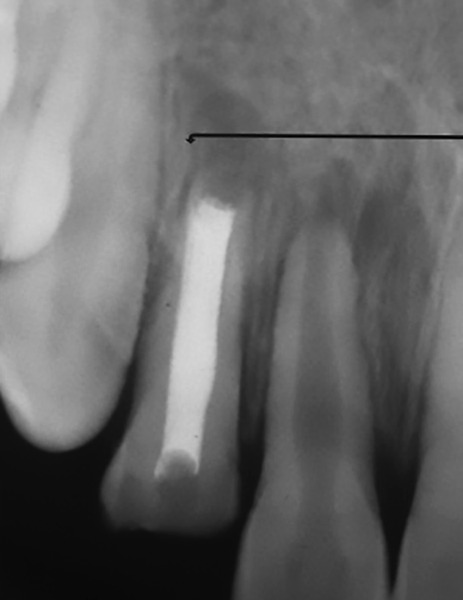
Intraoral periapical radiograph with an arrow showing retrograde filling done using zirconomer-reinforced glass ionomer cement and removal of root apex

Initially, while exploring the canal, a wooden green stick was found in the canal firmly stuck to the apex. He gave the history of putting the sticks in the canal since the fracture to counter irritation. Retrieval of that stick was tried using ultrasonic, H-Files, and ProTaper files, but all in vain. So, it was decided to treat it surgically. Labial mucoperiosteal flap was raised and bone cutting was done by surgical bur (Fig. 3). The displaced fractured immature root apex was removed along with the wooden stick (Fig. 4) and the retrograde filling was done using zirconium-reinforced glass ionomer cement (Fig. 5).

## DISCUSSION

Numerous cases of self-inflicted injuries leading to lodgment of foreign bodies have been reported. Retrieval of pencil tips, toothpick, and tomato seed has been reported by Grossman.^7^ These troublesome incidents take place due to carious exposure or traumatic injuries resulting in open pulp chamber. Food impaction into these pulp chamber further complicates the situation due to which children insert objects. These objects get wedged within the canal and may be pushed into the periapex. The exogenous material acts as a focus of infection and causes tissue irritation.

For retrieval of foreign objects in the pulp canal, many things that have been advocated like ultrasonic instruments, H files, etc., have been used.^8^ In our case also, the stick pushed in the periapex displaced the fractured immature root apex and stuck back, thus causing foreign body reaction. The reason for fracture of immature root apex was not known. It can be due to trauma 1 year back or due to the stuck stick in the apex region. Intra-canal medicament was not sufficient in our case due to stuck wooden stick, thus requiring apical surgery. The fractured immature root segment along with the stick acting as foreign body was removed and retrograde filling was done.

## CONCLUSION

Foreign bodies may be lodged inside the root canal because of open pulp chamber either due to caries or trauma. Most of the patients do not seek treatment as long as the tooth is asymptomatic. Proper counseling of the patient is required at the earliest to prevent any untoward incidents. Dental school camps should be organized to make them aware about the consequences and prevent any complications.
